# Mental Imagery and Post-Traumatic Stress Disorder: A Neuroimaging and Experimental Psychopathology Approach to Intrusive Memories of Trauma

**DOI:** 10.3389/fpsyt.2015.00104

**Published:** 2015-07-22

**Authors:** Ian A. Clark, Clare E. Mackay

**Affiliations:** ^1^Department of Psychiatry, University of Oxford, Oxford, UK

**Keywords:** post-traumatic stress disorder, mental imagery, intrusive memory, psychological trauma, experimental psychopathology, trauma film paradigm, neuroimaging, flashbacks

## Abstract

This hypothesis and theory paper presents a pragmatic framework to help bridge the clinical presentation and neuroscience of intrusive memories following psychological trauma. Intrusive memories are a hallmark symptom of post-traumatic stress disorder (PTSD). However, key questions, including those involving etiology, remain. In particular, we know little about the brain mechanisms involved in why only some moments of the trauma return as intrusive memories while others do not. We first present an overview of the patient experience of intrusive memories and the neuroimaging studies that have investigated intrusive memories in PTSD patients. Next, one mechanism of how to model intrusive memories in the laboratory, the trauma film paradigm, is examined. In particular, we focus on studies combining the trauma film paradigm with neuroimaging. Stemming from the clinical presentation and our current understanding of the processes involved in intrusive memories, we propose a framework in which an intrusive memory comprises five component parts; autobiographical (trauma) memory, involuntary recall, negative emotions, attention hijacking, and mental imagery. Each component part is considered in turn, both behaviorally and from a brain imaging perspective. A mapping of these five components onto our understanding of the brain is described. Unanswered questions that exist in our understanding of intrusive memories are considered using the proposed framework. Overall, we suggest that mental imagery is key to bridging the experience, memory, and intrusive recollection of the traumatic event. Further, we suggest that by considering the brain mechanisms involved in the component parts of an intrusive memory, in particular mental imagery, we may be able to aid the development of a firmer bridge between patients’ experiences of intrusive memories and the clinical neuroscience behind them.

## The Patient Experience of Intrusive Memories

I was in the car outside my house. The mugger put a knife to my neck; he said ‘give me your money’. I was scared he would realise that I live here; I was worried for my daughter. He then checked my pockets and asked for my purse to check it and rummaged through it. I was feeling helpless; I was worried I had forgotten some money and he would find it and say I was lying to him. He then ran off and I looked back to my house to see my daughter crying and banging at the door. I felt guilty that she may have seen what happened and that she would be traumatised by it.

A patient’s description of a traumatic event, taken from Holmes et al. (1).

Most people will experience or witness a traumatic event over the course of their lifetime and a significant minority will go on to develop post-traumatic stress disorder (PTSD) (2, 3). A hallmark symptom of PTSD is the experience of intrusive memories of the trauma (4). Clinically, intrusive memories are well documented. Our understanding of intrusive memories at a neuroscientific level on the other hand is not. Here, we present a pragmatic clinical-neuroscience framework for understanding intrusive memories, breaking intrusive memories into five component parts. We suggest that mental imagery is key to bridging the experience, memory, and intrusive recollection of the traumatic event. By understanding the individual components, and how mental imagery links each component together, we hope to be able to help bridge the gap between patients’ experiences as seen in the clinic and the clinical neuroscience behind them.

A traumatic event is defined not merely as a very stressful event but specifically as experiencing or witnessing serious injury or threat to the physical integrity to the self or others [Criterion A of the American Psychiatric Association (APA), Diagnostic and Statistical Manual 5 (DSM 5)] (4). This diagnostic criterion is particularly interesting as PTSD is one of the few disorders in the DSM 5 that requires an index event to have occurred for diagnosis. This opens up an area of investigation for clinical research to try to understand how PTSD arises from a specific event.

Not everyone who experiences a traumatic event develops PTSD. A diagnosis of PTSD requires four other types of symptoms in addition to experiencing psychological trauma. These are the hallmark symptoms of re-experiencing, including intrusive memories (Criterion B), persistent avoidance of trauma-related stimuli (Criterion C), persistent symptoms of increased arousal (Criterion D), and negative cognitions and mood (Criterion E), all of which need to be present for at least 1 month (Criterion F).

We focus here on the re-experiencing criterion of PTSD, specifically on intrusive memories. Most people experience intrusive memories after witnessing or experiencing a traumatic event, some of whom will go on to develop PTSD. Intrusive memories following trauma share many features between those individuals who do go on to develop PTSD and those who do not (5). Trauma can be re-experienced in different ways, all of which are highly distressing experiences. Intrusive memories are the spontaneous and repeated re-experiencing of the traumatic event, that is, involuntary images of the trauma intruding into consciousness (6). An example related to the patient description of a traumatic event described earlier [from Holmes et al. (1)] would be (1) the sudden image of the moment a mugger raised a knife, accompanied by an intense feeling of fear and (2) a separate image of her daughter’s crying face with the feeling of guilt.

Intrusive memories are rarely a replay of the entire traumatic event from beginning to end. Patients often recall one specific moment of the traumatic event at a time – known as a hotspot (7, 8). Hotspots are idiosyncratic – different individuals could witness the same trauma but have different hotspots that return to mind unbidden. They can also represent a range of different emotions that the individual experienced over the course of the trauma. The events in Table 1 are the hotspots of a different patient who was physically assaulted during a mugging. The hotspots depict a range of negative emotions, in this case, fear, humiliation, sadness, and degradation. On average, patients experience three to four hotspots per trauma, including emotions of fear, helplessness, anger, guilt, and shame (1, 9). These hotspots are those elements of the traumatic event that are re-experienced as intrusive memories.

**Table 1:** **Hotspots from one PTSD patient during a mugging**.

Event within trauma	Emotional reaction
Hands pulling at bag	They are trying to pull me over; Fear
Fallen down on the ground	I have lost, they have won, I am stupid; Humiliation
Kicked in stomach	They are taking away my chance to have children; Sadness
Assailants walking away slowly	They cannot even be bothered to run; Degraded

*Each hotspot is associated with specific emotions and meanings that are present when the images return as intrusive memories. Taken from Holmes et al. (1)*.

Not all experiences of trauma result in the persistent experience of intrusive memories and a diagnosis of PTSD. The question therefore arises as to why only some moments within a trauma are later experienced as intrusive memories. This is not a straightforward question to answer, particularly as traumatic events are difficult to study. Cognitive behavioral models of PTSD suggest that cognitive processing during the traumatic event has a large impact on the nature of the trauma memory (8, 10). Indeed, one of the strongest predictors of the development of PTSD is peritraumatic psychological processing (11), i.e., the individual’s experience during and immediately after the traumatic event – in particular, perceived life threat during the trauma, peritraumatic emotional responses, and peritraumatic dissociation. These processes are thought to affect the formation of the memory, the contextualization of the trauma within the experience, and subsequent appraisals of the event [see Ref. (8, 10), and neural models of PTSD and intrusive memories below]. Further, experimental studies suggest that peritraumatic psychological processes are also important for predicting intrusive memories following analog trauma [e.g., Ref. (12), see also “Intrusive Memories in the Laboratory” below]. The experience of the individual at the time of the trauma seems, therefore, to be important for predicting symptoms following trauma.

## Neural Models of PTSD and Intrusive Memories

Traditional neurocircuitry models of PTSD highlight the importance of three main brain regions; the amygdala, and its interactions with the ventromedial prefrontal cortex (vmPFC), and the hippocampus (13, 14). These models predominantly stem from animal work into fear conditioning, which has a number of parallels with PTSD symptomatology. Specifically, in response to threat-related stimuli, there is thought to be increased activation in the amygdala due to a diminished ability of the vmPFC and hippocampus to govern the amygdala responsiveness. Further, hyperactivity in the amygdala is proposed to explain the distinct emotional quality of memories of the trauma; hypo-response in the vmPFC the inability to move attention away from the trauma-related stimuli; and decreased hippocampal functionality that the poor voluntary recall patients’ show in regards to the traumatic event.

Neuroimaging studies in patients with PTSD show support for these neurocircuitry models. The symptom provocation paradigm has been widely used in neuroimaging studies to examine the brain activation occurring during the patient’s experience of PTSD symptoms, such as intrusive memories. The paradigm involves exposing individuals with PTSD to stimuli designed to trigger their symptoms, e.g., visual images of combat situations (15) or verbal autobiographical scripts of the patients’ trauma. Reviews of symptom provocation neuroimaging studies (16–18) suggest that PTSD patients’ symptom experience involves decreased activity of the anterior cingulate cortex (ACC), medial PFC, parahippocampus, and thalamus, and, generally, increased amygdala activity.

Further work suggests that abnormal interactions between the hippocampus and vmPFC may arise after developing PTSD, while abnormalities in the amygdala and dorsal ACC may be predisposing (19). However, while these structures may explain some elements of PTSD, it is unlikely that they alone can explain all symptoms associated with PTSD (20), in particular given the number of regions identified by symptom provocation studies. Thus, it is currently uncertain which of these brain regions may be associated directly with intrusive memories, and which others may be associated with, for example, increases in arousal.

A distinct model of intrusive memories stems from clinical psychology and the neuroscience of memory. Brewin et al. (21), see also Ref. (22), suggest that there are two forms of memory representations – those that are abstract and contextually bound, and those that are sensory and affective in nature and not contextually bound. In a healthy memory, these two representations are connected. An intrusive memory on the other hand has a strong sensory representation that is not connected to its contextual representation. This allows the memory to be easily cued by trauma-related information and without any autobiographical context – creating the re-experiencing feelings common to intrusive memories. Relating these concepts to neural mechanisms, Brewin et al. suggest, in line with neurocircuitry models of PTSD, that intrusive memories occur due to hyper-activation in the amygdala and insula, which is disconnected from the hippocampus and related memory structures that are required to provide contextual autobiographical information. Coupled with visual imagery (suggested to be mediated by the precuneus), the intrusive memory then appears involuntarily in mind as a visual memory.

Support for these suggestions also stems from patient studies investigating intrusive memories directly. Only a small number of studies have been able to investigate the explicit occurrence of intrusive memories. The symptom provocation paradigm does not always cause patients to experience intrusive memories. The paradigm serves as a reminder of the trauma, bringing trauma memories to mind, causing, for example, heighted emotional responses and avoidance, but does not necessarily cause involuntary intrusive memories. To our knowledge, only four neuroimaging studies of PTSD have explicitly reported the brain activation of patients experiencing “flashbacks” while undergoing symptom provocation (23–26). These studies suggest that the experience of an intrusive memory may involve increased activity in limbic and paralimbic areas including the insula, ACC, thalamus, and amygdala, and decreased activation in inferior frontal areas – presenting clues as to those regions that may be involved in intrusive memories specifically. While it should be noted that these studies did not capture the *moment* of intrusive memory involuntary recall, but rather the more general experience surrounding intrusive memories, they do share similarities with the neural mechanisms proposed to underlie intrusive memories (13, 14, 21).

The above-mentioned neuroimaging studies all examined brain activation in patients once symptoms are already established. Key questions including those involving etiology nevertheless remain. For example, why do some people experience intrusive memories and not others? Why do certain moments of the original trauma return as intrusive memories but not others? While it is not possible to investigate the brain mechanisms behind symptom development during real trauma, analog models may offer a prospective methodology to investigate the etiology of intrusive memories.

## Intrusive Memories in the Laboratory

**Figure 1:**
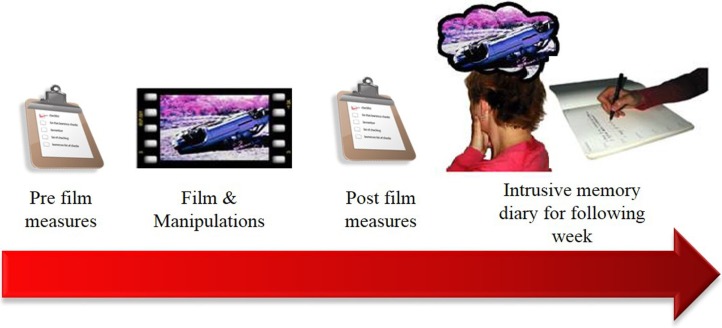
**Diagram of the general procedure of the trauma film paradigm**. Participants view a distressing film as an analog of a traumatic event. Over the following week they record any intrusive memories of the film in a diary. This allows for investigation of baseline differences affecting intrusive memory development, or tasks that might increase/decrease later intrusive memories.

Real life traumatic events and the subsequent development of intrusive memories are difficult to study in laboratory settings due to both ethical and practical reasons. The trauma film paradigm (Figure 1) is a well-established method to provide an analog model to prospectively investigate intrusive memories in controlled laboratory settings (27–29). In the paradigm, healthy participants watch a film depicting traumatic events, such as the aftermath of real life car crashes. Participants typically experience several intrusive memories to events in the film during the following week, operationalized to participants as: (1) moments of the film spontaneously popping into mind unexpectedly and (2) mental images, i.e., taking the form of pictures, sounds, or bodily sensations. These intrusive memories are recorded in a diary, similar to diaries given to PTSD patients undergoing CBT. The majority of participants experience at least 1 intrusive memory to events in the film, with an average frequency (from 16 studies, totaling 458 participants) of around 5–6 (12). Information recorded in the diary allows for features of the intrusive memory (e.g., number, vividness, emotional rating) to be recorded as well as identification of the film scene (analog hotspot) that the memory originated from.

We note that the trauma film paradigm is an analog methodology and not the same as experiencing real life trauma. While findings are preliminary, repeated exposure to electronic media images of the September 11th terrorist attacks in 2001 have been associated with measures of PTSD symptoms 2–3 years later, though predominantly for those individuals viewing >4 h a day for the week following the attack (30). Further, individuals repeatedly exposed to media footage of the 2013 Boston marathon bombings (6 h a day for the week following the bombings) reported higher acute stress symptoms 2–4 weeks later than those directly exposed to the event (31). While the DSM 5 has also acknowledged within the PTSD diagnosis that exposure to trauma images through electronic media, television, and movies in the line of work (4) can be sufficient to lead to PTSD, the full relationship between media exposure and PTSD symptoms is unclear. Regardless, given the potential for electronic media images to cause symptoms, understanding PTSD symptom development from electronic media images of trauma remains pertinent, in addition to being able to inform on real life symptom development.

The trauma film paradigm has allowed for detailed investigation into peritraumatic factors that may affect symptom development, which can be more difficult in clinical research studies due to data often being collected retrospectively. For example, research has shown that performing visuospatial tasks during, or soon after, exposure to analog trauma can reduce intrusive memory frequency [e.g., Ref. (28, 32)]. Further, changes in state anxiety (33) and emotional processing (12) in response to film viewing have been associated with intrusive memory frequency, as well as possible vulnerability factors, e.g., attentional control (34). The trauma film paradigm therefore offers an opportunity to investigate the development of intrusive memories in controlled settings, in particular, peritraumatic factors. The trauma film paradigm therefore opened up a possible mechanism to understand the brain mechanisms involved in intrusive memory formation.

## Neuroimaging the Encoding of Analog Intrusive Memories

To our knowledge, only two studies have used neuroimaging to investigate the encoding of emotional images during a trauma film that participants later re-experience as intrusive memories (35, Clark et al., under review).

Bourne et al. (35) conducted the first study implementing the trauma film paradigm to examine the differences in brain activations when viewing “Potential scenes” (unpleasant scenes that elicited intrusive memories in other participants but not in that participant), with “Intrusive (referred to as Flashback) scenes” (those unpleasant scenes that did elicit intrusive memories). Results suggested a widespread neural signature at the time of viewing those scenes that would later be re-experienced as intrusive memories including increased activation in the amygdala, thalamus, rostral ACC, striatum, and ventral occipital cortex. Additionally, two regions seemed to distinguish between intrusive scenes and potential scenes: the left inferior frontal gyrus and middle temporal gyrus.

Given potential limitations and difficulties in studying the neural basis of rare idiosyncratic events, such as intrusive memories (e.g., low event count), replication of these results was important. We therefore conducted a second experiment using an independent sample finding an almost exact replication of our previous results (Clark et al., under review). Additionally, using multivariate pattern analysis techniques, we were also able to predict later intrusive memory occurrence solely from the brain activity at the time of viewing traumatic footage (36). Thus, these results suggest that, at the time of trauma, the brain is responding differently to those scenes that later become intrusive memories compared to those scenes that do not cause intrusive memories for that individual. Given the widespread nature of these activations, it is important to understand how these regions may be involved in the formation of an intrusive memory. That is, what is it that makes the combination of these activations lead to the later involuntary re-experiencing of that specific event during psychological trauma?

## A Clinical-Neuroscience Framework of Intrusive Memories

We therefore aim to build upon previous theories and research into the underlying neural mechanisms of intrusive memories. Current neural theories suggest that intrusive memories occur due to poor integration of the trauma into memory (21). Given the proposed parallels between fear conditioning and PTSD (13), it is also possible that the brain processes involved in fear conditioning contribute directly to intrusive memory formation. We suggest that further cognitive processes in addition to fear conditioning may also be involved in intrusive memory formation and recollection. For example, intrusive memories are not purely fear based, involving multiple other emotions, for example, helplessness, anger, guilt, and shame (1, 9) – a fear conditioning account may therefore be only able to explain some of the underlying mechanisms. Brewin et al. (21) highlight disrupted autobiographical memory encoding, in combination with heightened emotional processing and mental imagery, yet our recent neuroimaging work investigating analog intrusive memory formation (35, Clark et al., under review) suggests, in contrast, heightened involvement of memory-related areas in addition to emotional processing and mental imagery. As such, we propose a pragmatic clinical-neuroscience framework of intrusive memories taking intrusive memories as part of a continuum of normal functioning. We suggest that by looking at intrusive memories as a combination of non-clinical cognitive processes that have been researched outside of the clinical literature in detail, we can use knowledge of these areas to help inform our understanding of the mechanisms behind intrusive memories.

**Figure 2:**
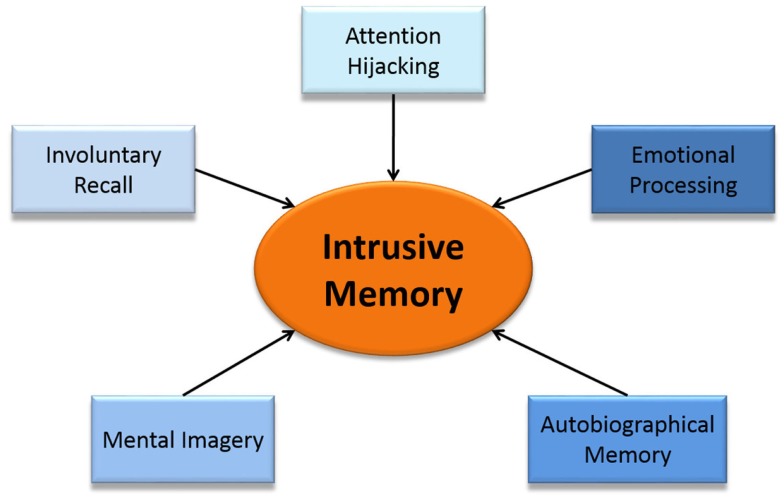
**A proposed clinical-neuroscience framework of intrusive memories breaking intrusive memories into five component parts**.

**Figure 3:**
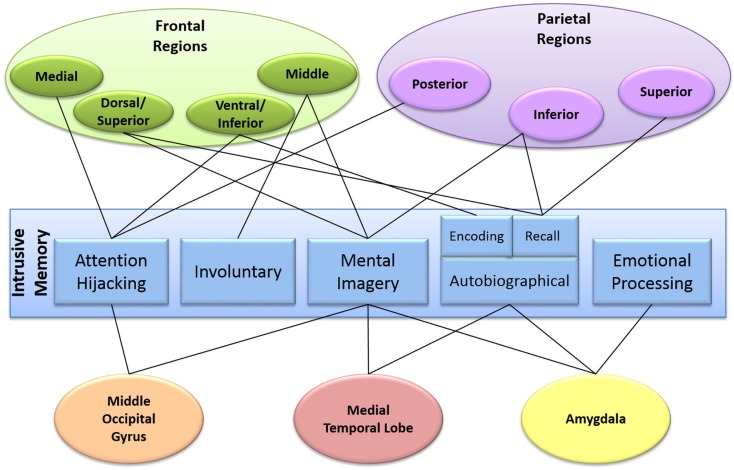
**Diagram mapping the patient experience of an intrusive memory onto the brain**. The components of our clinical-neuroscience framework are in the center, surrounded by the different brain areas involved.

Cognitive models and clinical descriptions suggest that intrusive memories are sensory–perceptual (predominantly visual) emotional memories of traumatic events that intrude involuntarily into consciousness, hijacking current selective attention (4, 8, 21, 22, 37, 38). We therefore divide intrusive memories into five component parts; autobiographical (trauma) memory, involuntary recall, emotional processing, attention hijacking, and mental imagery (Figure 2). We hypothesize that heighted involvement of each of these cognitive processes are involved in the underlying mechanisms of the formation and experience of an intrusive memory. In the following sections, we review each component in terms of its everyday cognitive process, suggest how each of these five components are involved in intrusive memories and briefly summarize what is known of the neural components behind them. We then map the patient experience of an intrusive memory onto the brain, culminating in Figure 3, with the components of our clinical-neuroscience framework in the center surrounded by the different brain areas involved.

## Autobiographical (Trauma) Memory

An autobiographical memory is a personal memory that either corresponds to a particular episode in life, or to a more general experience that has particular personal relevance (39). The autobiographical component of an intrusive memory corresponds to the personal experiencing (Criterion A) and subsequent re-experiencing (Criterion B) of the traumatic event, as set out in the DSM 5 (4). The importance of autobiographical memory for intrusive memories is acknowledged in cognitive and clinical theories of PTSD (8, 21, 38).

The literature surrounding autobiographical memory is vast; for recent reviews on the neuroimaging of memory, see Cabeza and Nyberg (40) and Spaniol et al. (41). Overall, these reviews conclude that autobiographical memory is normally associated with activity in the right anterior and lateral prefrontal gyri, the medial temporal lobe, the lateral and medial parietal regions, and the posterior cingulate cortex. Specifically, the ventrolateral prefrontal gyrus and medial temporal lobes are thought to be involved in encoding, while the left superior parietal gyrus and the dorsolateral and anterior prefrontal gyrus are thought to play an important role in recall (41–43). The “subsequent memory effect” suggests that activity at encoding in left prefrontal and bilateral middle temporal regions can predict later successful memory recall (44, 45). Additionally, it is thought that the full encoding of a memory takes place in a 6-h window (46, 47), a process known as consolidation. Research has implicated the involvement of the hippocampus in particular, but cortical areas including the nucleus accumbens and ventral striatum are also proposed to be involved (48).

## Involuntary Recall

Involuntary recall is the return of a mental state that was once present in consciousness with apparent spontaneity and without any act of will or previous attempts at retrieval (49–51). Intrusive memories come to mind spontaneously and in an unbidden manner, and are, therefore, recalled involuntarily. Thus, it is important to understand how the traumatic event is involuntarily, as opposed to voluntarily or deliberately, recalled as is often researched within autobiographical memory.

Behavioral and cognitive differences between involuntary and voluntary recall have been widely reported on. For example, involuntary recall is characterized by shorter retrieval times (52) and involuntary memories are more often of specific episodes than deliberately recalled memories [(32, 53), see also Ref. (54, 55)]. However, to our knowledge, there are only a handful of neuroimaging studies that have directly compared voluntary and involuntary recall (56–60). Further, only one study showed increased activation during involuntary compared to voluntary recall – in the left middle frontal gyrus and left superior frontal gyrus (57). On the other hand, greater activation for voluntary versus involuntary recall has been found in the right dorsolateral frontal cortex and parietal cortex (56, 58), in the right middle frontal gyrus (57), the left dorsolateral PFC (61), and the hippocampus and amygdala (59). Overall, these studies suggest that while both involuntary recall and voluntary recall activate regions associated with autobiographical memory, voluntary recall additionally activates areas associated with strategic recall. However, it remains to be established whether these findings can also be generalized to intrusive memory involuntary recall, and further replication of regions associated with involuntary over voluntary recall is required.

## Emotional Processing

Emotion is a subjective, conscious experience characterized by biological reactions, and an individual’s current mental state. The patient experience of intrusive memories is characterized by strong negative emotions. Emotional processing at the time of trauma has also been highlighted as important for later PTSD development (11) and for intrusive memories following analog trauma (12). Hyperactivity of emotional regions has also previously been proposed to be important in both PTSD and intrusive memory development (13, 21).

It is important to define these emotions as negative when investigating intrusive memories experimentally as involuntary memories are not always distressing. Research has found that involuntary memories are not limited to negative experiences or indeed to clinical populations – a telephone survey of 1500 Danes identified that approximately 60% of involuntary memories reported were positive in nature (62). Additionally, positive involuntary memories in the laboratory have also been associated with increased (positive) emotional processing at the time of encoding (63). Behaviorally and clinically, intrusive memories are regarded as negative, whether a distinction between negative and positive emotion is required, however, is less clear in terms of the mechanisms underlying intrusive (or involuntary) memories.

At a neural level, research into emotion often implicates the amygdala, ACC, and the PFC (64–66). The amygdala is traditionally associated with negative emotions, especially that of fear (66, 67). However, more recent work suggests that the amygdala is also involved in positive emotions (68) and that the amygdala may respond to emotional salience rather than to whether the emotion is positive or negative (69). Indeed, amygdala activation at encoding has been associated with success of recall regardless of emotional valence (70, 71). Additionally, the ACC is often implicated in threat detection, and the PFC is thought to be involved in emotion regulation – allowing top down control in response to emotional situations (64, 72). At a neural level therefore, emotional valence may be less important than the intensity of the experienced emotion, i.e., how emotionally salient the event was, or there may be a down play in top down control of emotion regulation.

## Attention Hijacking

Sensory information in the world around us is abundant, and attention is used to select the information that is relevant at a given time (73). Attention hijacking is the overriding of this selective attention geared toward our current goal, transferring attention to something else. To become salient, an intrusive memory must hijack attention to some degree.

How might an intrusive memory override selective attention? Research suggests that PTSD patients have enhanced priming for perceptual and verbal trauma-related stimuli (74, 75). Measurement of this enhanced priming soon after trauma was also associated with symptom severity at later follow-ups. Comparison of PTSD patients and trauma-exposed controls suggests that enhanced priming for trauma-related stimuli may be an inability of patients to move their attention away from trauma-related stimuli and not an increase of facilitated attention to trauma-related stimuli (76, 77). A poor ability to remove information that is no longer relevant from mind, measured in non-clinical participants before viewing traumatic footage, has also been associated with intrusive memory frequency in the subsequent week (34, 78). Overall, this work suggests that an inability to move attention away from non-relevant stimuli may be a vulnerability factor for intrusive memory development. However, this work has focused upon external trauma-related stimuli, and not internal representations as per an intrusive memory. Notably, there are a number of similarities between attention toward internal and external representations, in particular, in terms of behavioral responses (79, 80). Thus, it may be possible to extrapolate the above trauma-related findings to internal as well as external representations.

In healthy individuals, visual selective attention has been associated with activity in widespread brain regions, including the parietal, temporal, and prefrontal cortices (81). It has been proposed that the frontal regions deal with specifying, consolidating, and selecting targets, while the posterior parietal, occipital, and temporal regions filter out distracting stimuli (82–84). Investigations into attention toward internal representations have shown similar patterns of activation between internal and external stimuli but with some notable differences – right inferior parietal cortex was selectively important for attention toward external stimuli, while the frontal regions (in particular left inferior frontal gyrus) were selectively important for internal stimuli (80, 85). Our work investigating intrusive memory encoding (35, Clark et al., under review) highlights possible distinguishing activity in the left inferior frontal gyrus for those moments that will later become intrusive memories compared to those that will not. The left inferior frontal gyrus has been associated with the selection of information (86) and the “flexibility” to switch from one task to another (87) and thus may represent attention hijacking within intrusive memories, but this remains to be further explored.

Neuroimaging of attention in PTSD patients has shown decreased activity in PTSD patients with high levels of symptomatology compared to low symptomatology in dorsolateral PFC and parietal regions for neutral targets, but increases in these regions for emotional distractors. Additionally, in contrast to above, bilateral inferior frontal gyrus activity was higher in patients with low symptoms than those with high symptoms in response to the emotional distractor stimuli (88). Further, attention toward emotional distractors has been associated with the dorsolateral and ventral PFC (89). Using non-emotional stimuli, research has suggested a general hyper-vigilance of PTSD patients with increased activation of somatosensory and posterior parietal attention networks, inferior frontal gyrus and vmPFC, dorsal ACC, and amygdala (90). To our knowledge, however, attention toward internal stimuli in patients with PTSD has not yet been investigated.

## Mental Imagery

Mental imagery is a quasi-perceptual experience, in that it resembles perception and sensory experiences, but occurs in the absence of the appropriate perceptual and sensory stimuli (91, 92). There are arguments that intrusive memories in PTSD are not limited to sensory images, also including abstract thoughts; however, these types of “intrusions” are more like rumination and not what we aim to explain here [see also Ref. (93)]. Rather, we focus upon the more common experience of intrusive memories as sensory-based images (1, 94, 95). Mental imagery is highly connected to emotion, causing the same emotional responses as seeing an object or event itself (96). Additionally, mental imagery has been reported to have a similar effect on the body as actually seeing the object or event in question – skin conductance, heart, and breathing rate all increase when visualizing threatening objects (97). Indeed, mental imagery can also be confused with reality (96). Thus, given also that emotional memories are thought to be perceptual in nature (98), mental imagery may be an overarching component in why intrusive memories are such distressing experiences.

Neuroimaging investigations of mental imagery support the links between mental imagery, memory, emotion, and perception. First, neuroimaging research has consistently reported activation of the visual cortex during visual mental imagery (99). Additionally, visual mental imagery has been found to activate the middle frontal gyrus, the superior frontal gyrus, the middle occipital cortex, right ACC, and the left inferior parietal cortex (100), along with the hippocampus, amygdala, entorhinal cortex, parahippocampal cortex, and the anterior insula (101, 102). Thus, the experience of mental imagery additionally activates those regions previously associated with autobiographical memory and emotional processing as well as visual processing. Mental imagery may therefore be key to linking the other components of our intrusive memory framework.

We note here that the above evidence is focused upon visual mental imagery. Visual intrusions are the most common following trauma and are those that are typically studied in experimental settings; however, intrusive memories can be in other sensory modalities (e.g., audition and bodily sensations), physiological or manifest in other behavioral ways (93). On the other hand, given the connections between mental imagery and physiological arousal and related factors, it is likely that mental imagery plays a key role in all forms of intrusive recollections. Further, we suggest that while changing from visual to, for example, auditory mental imagery would change some of the brain regions activated (e.g., from visual cortex to primary auditory cortex), other underlying mechanisms would remain the same.

## Mapping the Experience of an Intrusive Memory Onto the Brain

Using our clinical-neuroscience framework, we hypothesize that a series of events happen simultaneously to create an intrusive memory (Figure 2). Neural mechanisms involved in involuntary recall activate the autobiographical (trauma) memory, which hijacks current selective attention. The mental imagery of the autobiographical memory, which is activated by the involuntary recall, intensifies the emotion of the event increasing the strength and attention hijacking nature of the intrusive memory.

Figure 3 maps our clinical-neuroscience framework onto the brain. The center of the diagram shows the different components that make up an intrusive memory. Surrounding this are the brain regions proposed to be involved. As can be seen, intrusive memories are a whole brain phenomenon with many areas involved in multiple components of the intrusive memory framework. Additionally, many of the proposed brain regions identified in each of the components (with the exception of involuntary recall) were those also observed during intrusive memory encoding (35, 36 Clark et al., under review).

What, therefore, can be seen by mapping the hypothesized components onto the brain? Interestingly, the brain areas involved in mental imagery are also involved in the other proposed components. For example, in addition to mental imagery, the frontal areas of the brain have been associated with attention hijacking and involuntary recall, suggesting that the frontal regions may be involved in the spontaneous recollection of mental images. Areas associated with autobiographical memory (predominantly the medial temporal lobe, but also parietal and frontal regions) have also been found to be involved in mental imagery, demonstrating a possible connection between the traumatic memory and mental imagery. Parietal regions, in addition to autobiographical memory are also involved in attention hijacking, suggesting a link between the traumatic memory and an overriding of attention. Amygdala activation is predominantly associated with emotional processing but also mental imagery and autobiographical memory, linking the emotional response and the mental image of the traumatic memory. Thus, there are connections between all five components of our hypothesized model of intrusive memories, linking back to mental imagery. *Combining each of these individual components via mental imagery, which on their own are required for normal functioning, may lead to the experience of an intrusive memory*.

## Building upon Previous Models of Intrusive Memories

How does this help us? What do we gain by looking at intrusive memories of trauma in this way? Much more work is needed to understand intrusive memories, especially in terms of neuroscience (50). Understanding how each component individually contributes to intrusive memories, and the neuroscience behind it, increases our knowledge of an otherwise incredibly complex phenomenon. While our understanding of the neuroscience behind intrusive memories *per se* is limited, much more is known about the neuroscience of autobiographical memory, emotion, attention, and mental imagery outside of the clinical literature.

Further, the current model differs to previous models [e.g., Ref. (21, 22)] in that we suggest that *heightened* memory processing, not disrupted processing, may be important for intrusive memory formation. Additionally, we include a separate component of attention hijacking. By doing so, we highlight the possible involvement of more frontal regions of the brain in addition to the subcortical regions noted by Brewin et al. (21). We also note the overarching connection between each of the components that make up an intrusive memory – that of mental imagery. While imagery has often been associated with intrusive memories, our framework suggests that it may be key in uniting all aspects of an intrusive memory.

## Predictions and Testable Hypotheses

By tailoring research in areas identified by our framework toward intrusive memories and PTSD, we may be able to develop a better clinical-neuroscience understanding of the patient’s experience of intrusive memories. Further, by breaking intrusive memories into our proposed components, we have a framework for developing and testing hypotheses.

We hypothesize that mental imagery is an overarching component of intrusive memories that has links to all of the other components – while each of the other components may overlap with others, mental imagery is the only one to unite all of the components. By removing mental imagery, or occupying mental imagery processes, we may be able to directly affect the formation and involuntary recall of intrusive memories. For example, work using the visuospatial computer game Tetris has already shown its ability to reduce visual intrusive memory frequency in experimental settings (32, 103, 104). Further, a high tendency or ability to use mental imagery may be an important risk factor in intrusive memory development [e.g., Ref. (105)]. Understanding how mental imagery relates to real world trauma may help develop easy to administer screening measures for at-risk populations.

Additionally, mental imagery is also a broad term used to encompass all aspects of perceptual information accessed from memory (92). Intrusive memories do not have to be visual in nature – some patients experience intrusive memories in the form of audition or bodily sensations. A more precise understanding of the specific aspects of mental imagery associated with intrusive memories may further refine potential treatments. Adaptation of imagery-based therapies to other modalities may be important to address other types of intrusions. Further, while we suggest that the underlying mechanisms involved in intrusive memories of these different modalities may be similar to that of visual intrusions, there may also be important differences between these types of intrusive recollections that should be investigated in future work.

We also suggest the possible importance of heightened memory processing during exposure to moments of the trauma that later return as intrusive memories (see also, 35, 36, Clark et al., under review). This is in contrast to other clinical cognitive neuroscience models of intrusive memories (22), but a proposal that has parallels with memory-based models of PTSD (38). Understanding exactly how memory processes contribute to intrusive memory formation and recall is essential for future work into the neural basis of intrusive memories.

The proposed framework also suggests that we may be able to disentangle emotional processing and the memory of the trauma. The experience and subsequent memory of the trauma is required for an intrusive memory, but the emotional reaction is what makes the intrusive memory distressing for patients. Is it therefore possible to reduce the emotion associated with the intrusive memory, while keeping the memory itself intact? Additionally, how can we go about doing this at a neural level? The overarching aim of treatment is to reduce the distress associated with intrusive memories – removing the memory itself is not necessarily the best response (106).

In addition to the negative emotions brought to mind by intrusive memories, their involuntary nature and hijacking of attention further exacerbate the distressing effects. Establishing and understanding possible neural differences between voluntary and involuntary autobiographical memory recall may help pinpoint areas of memory that are distinct to intrusive memories. From this, possible ways of reducing the frequency of involuntary recall of these memories may become further apparent.

The neural processes of attention suggest an important role for frontal regions in allowing/enabling the intrusive memory to come to the forefront of selective attention. Typically, decreased ventral–medial PFC activity is associated with PTSD, which is thought to reduce the control of the amygdala, heightening emotional responses. However, the PFC may also be important in the attention hijacking nature of intrusive memories, or toward external stimuli that then trigger intrusive memories. Further research is required to disentangle the role of attention in intrusive memories. Understanding how an intrusive memory overrides selective attention may present clues as to reducing intrusive memory impact at involuntary recall.

Finally, the neural basis of the encoding of intrusive memories is an area that has received only recent attention. Further work to understand the identified neural signature and how the brain activity relates to underlying cognitive processes will help in translating findings toward possible treatment development.

## Discussion and Conclusion

We set out here a clinical-neuroscience framework that considers intrusive memories as a combination of five component parts: autobiographical (trauma) memory, involuntary recall, negative emotions, attention hijacking, and mental imagery (Figure 2). Our clinical-neuroscience framework aims to set out some experimental hypotheses for mapping the brain processes that contribute to the experience of intrusive memories. Intrusive memories are a highly complex phenomenon – by considering them as a combination of component parts, which individually have received substantial research, we hope to suggest alternative hypotheses that may otherwise go overlooked.

The need to bridge the gap between neuroscience and mental illness is becoming increasingly recognized as a necessity for continued improvement of psychological therapies [e.g., Ref. (107–109)]. Understanding mechanisms, both at the cognitive and neural level, behind both symptom development and symptom experience may go some way to help increase treatment efficacy. Neuroscience provides a potential tool to help improve understanding of psychological symptoms. Our clinical-neuroscience framework of intrusive memories presented here presents additional steps to help bridge neuroscience and the presentation of intrusive memories, demonstrating the possibilities of combining these two disparate areas.

Overall, we suggest that mental imagery may be key to the formation and experience of an intrusive memory. Mapping the neural correlates of the five component parts together (Figure 3) suggests that mental imagery may be involved in combining the components into the experience of an intrusive memory. That is, mental imagery may bridge the experience, memory, emotional processing, attentional hijacking, and intrusive recollection of the traumatic event. By understanding the contribution of mental imagery in particular to the development of intrusive memories, we hope to be able to build a firmer bridge between patient’s experiences and their psychological and neuroimaging underpinnings.

## Conflict of Interest Statement

The Review Editor Anke Ehlers declares that, despite being affiliated to the same institution as authors Ian A. Clark and Clare E. Mackay, the review process was handled objectively and no conflict of interest exists. The authors declare that the research was conducted in the absence of any commercial or financial relationships that could be construed as a potential conflict of interest.
